# Nursing intensity trajectory patterns and clinical outcomes in intensive care units: a latent class analysis

**DOI:** 10.1016/j.ijnsa.2026.100613

**Published:** 2026-06-26

**Authors:** Qinqin Shu, Yan Li

**Affiliations:** Department of Emergency Medicine, Shanghai Fourth People's Hospital, School of Medicine, Tongji University, 1279 Sanmen Road, Hongkou District, Shanghai, 200434, China

**Keywords:** Intensive care, Nursing intensity, Trajectory analysis, Latent class analysis, Patient outcomes, SOFA score

## Abstract

**Background:**

Temporal patterns of nursing intensity during ICU stay may identify clinically meaningful patient subgroups, yet existing studies rely primarily on static measurements at single time points.

**Objectives:**

To identify distinct nursing intensity trajectory patterns during the first 7 ICU days and examine their associations with hospital mortality and ICU length of stay, independent of baseline illness severity.

**Design:**

Retrospective cohort study using latent class trajectory modeling. This study was not prospectively registered as it involved retrospective analysis of an existing database.

**Setting:**

Medical and surgical intensive care units at Beth Israel Deaconess Medical Center (2008–2019).

**Participants:**

7,334 adult ICU patients (≥18 years) with ICU length of stay ≥7 days.

**Methods:**

Nursing intensity was measured daily using a composite score incorporating three dimensions: sedation management (RASS scores), monitoring frequency (nursing charting counts), and care complexity (organ support requirements). Latent class trajectory modeling identified distinct patterns over ICU Days 1–7. Multivariable logistic regression examined associations with hospital mortality, adjusting for age, sex, and SOFA score.

**Results:**

Four distinct trajectory classes were identified (entropy = 0.948): Class 1 ‘Rapid Improvement’ (n = 702, 9.6%) showed steeply declining NI from 56.2 to 29.8, with the lowest mortality (9.6%) and shortest ICU stay (median 8.7 days). Class 2 ‘Late Escalation’ (n = 360, 4.9%) showed rising NI from 38.5 to 61.7, with the highest mortality (39.3%) and longest stay (median 16.6 days). Class 3 ‘Moderate Stable’ (n = 1,798, 24.5%) and Class 4 ‘Persistent High’ (n = 4,474, 61.0%) showed intermediate outcomes. After adjusting for age, sex, and SOFA, Rapid Improvement was associated with 74% lower mortality odds (OR = 0.26, 95% CI: 0.18–0.36, p < 0.001) and Late Escalation with 86% higher odds (OR = 1.86, 95% CI: 1.45–2.39, p < 0.001) compared to Persistent High. Sensitivity analysis excluding medical intervention variables confirmed trajectory robustness (Adjusted Rand Index = 0.565). Bootstrap validation (500 resamples) confirmed class stability.

**Conclusions:**

Four distinct nursing intensity trajectory patterns were identified, each characterized by different clinical outcome profiles. Declining NI trajectories characterized patients with lower mortality and shorter ICU stays, while escalating trajectories were observed in patients with worse outcomes, independent of baseline illness severity. These observational findings are hypothesis-generating and require prospective validation before informing clinical practice.

**What is already known about the topic?**

• Higher nurse-to-patient ratios and intensive monitoring are traditionally assumed to improve ICU outcomes uniformly for all critically ill patients.
• Illness severity scores such as SOFA predict mortality but do not capture dynamic patterns of care intensity delivery over time.
• Trajectory-based analyses have identified clinically meaningful patient phenotypes in other critical care contexts, but have not been applied to nursing intensity patterns.

Alt-text: Unlabelled box dummy alt text

**What this paper adds**

• Identifies four distinct nursing intensity trajectory patterns during the first 7 ICU days, each with different clinical outcome profiles.
• Demonstrates that trajectory class membership is associated with hospital mortality independent of baseline illness severity (SOFA score), suggesting these patterns capture clinically relevant information beyond severity alone.
• Provides empirical foundation for future prospective research on whether early identification of trajectory patterns could inform individualized nursing care intensity approaches.

Alt-text: Unlabelled box dummy alt text

## 1 Introduction

### 1.1 Background

Intensive care units (ICUs) represent the highest level of nursing intensity in modern healthcare, where critically ill patients require constant monitoring and complex interventions. Traditional ICU nursing models have generally operated under the assumption that higher care intensity—through frequent monitoring, active intervention, and therapeutic escalation—is associated with improved patient outcomes (Aiken et al., 2002; Driscoll et al., 2018; Ross et al., 2023). This framework has substantially influenced staffing models, resource allocation, and quality metrics (Twigg et al., 2021).

However, the relationship between nursing intensity and outcomes may be more nuanced than a simple dose-response model suggests. Iatrogenic complications from intensive intervention—including delirium from prolonged sedation (Barr et al., 2013; Ely et al., 2004; Patel et al., 2014), healthcare-associated infections from invasive monitoring, and ventilator-associated complications—raise questions about whether uniformly high-intensity care is optimal for all patients (Schweickert et al., 2009). Furthermore, research on physiological variability suggests that preserved adaptive capacity may be a marker of health in critical illness (Buchman, 2002; Goldberger et al., 2002; Seely and Macklem, 2004).

### 1.2 Conceptual framework: trajectory patterns in critical care

We propose examining nursing intensity not as a static characteristic measured at a single time point, but as a dynamic trajectory that may identify distinct patient subgroups with different clinical courses (Nagin and Odgers, 2010; Seymour et al., 2019). During an ICU stay, patients may exhibit varying temporal patterns of care intensity—some improving rapidly with declining care needs, others requiring persistently high or escalating support. Understanding whether these trajectory patterns are associated with clinical outcomes, independent of initial illness severity, could inform hypothesis generation for future studies of individualized care approaches (Curley, 1998).

Importantly, these trajectory patterns are descriptive constructs reflecting the interplay between patient physiology and care delivery, rather than normative judgments about care quality or causal claims about the effects of care intensity.

### 1.3 Knowledge gaps

Despite extensive research on nursing intensity and patient outcomes, critical knowledge gaps remain. First, existing studies have primarily used static measurements at single time points, failing to capture temporal patterns that may have prognostic significance. Second, the relationship between nursing intensity patterns and outcomes has not been examined independent of baseline illness severity. Third, whether declining nursing intensity trajectories are associated with better or worse outcomes remains empirically unknown. Fourth, the relationship between nursing intensity trajectory patterns and ICU length of stay has not been formally analyzed.

### 1.4 Study aims

This study addresses these gaps through three specific aims:

Aim 1: Identify distinct nursing intensity trajectory patterns during the first 7 ICU days using latent class trajectory modeling.
Aim 2: Characterize the clinical features, illness severity, and ICU length of stay associated with different trajectory patterns.
Aim 3: Determine associations between trajectory pattern membership and hospital mortality after adjustment for baseline illness severity using the SOFA score.

## 2 Methods

### 2.1 Design and setting

We conducted a retrospective cohort study using data from the Medical Information Mart for Intensive Care IV (MIMIC-IV) database version 2.2 (Johnson et al., 2023), containing deidentified health data from patients admitted to ICUs at Beth Israel Deaconess Medical Center in Boston, Massachusetts, between 2008 and 2019. Beth Israel Deaconess Medical Center is a 673-bed tertiary academic medical center affiliated with Harvard Medical School and a Level I trauma center. The study was exempted from institutional review board approval due to the deidentified nature of the data. Data access was obtained through completion of the Collaborative Institutional Training Initiative (CITI) program.

To our knowledge, AI-assisted ICU control center technology was not deployed at Beth Israel Deaconess Medical Center during the study period (2008–2019). The study period includes December 2019, during which undetected SARS-CoV-2 infections may have been present in the US population, including Massachusetts (Basavaraju et al., 2020). However, given the rarity of such cases before widespread community transmission and the 11-year study period, any influence on our findings would be negligible.

### 2.2 Study population

**Fig. 1:**
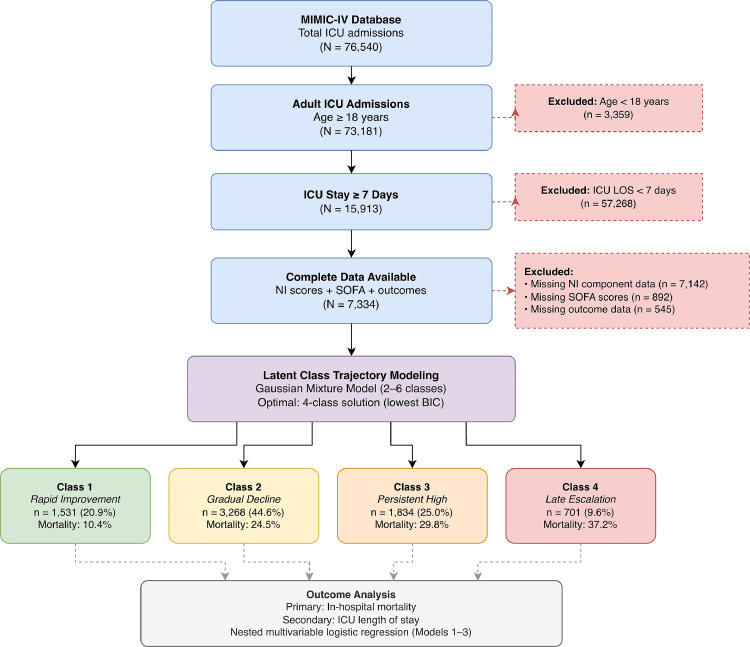
Patient selection flowchart (STROBE). Starting from 76,540 MIMIC-IV ICU admissions (2008–2019), patients were sequentially excluded based on age <18 years (n = 3,359), ICU length of stay <24 hours (n = 57,268), ICU length of stay <7 days (n = 8,579), and missing covariates or outcomes (n = 0). The final cohort of 7,334 patients was classified into four trajectory classes by latent class trajectory modeling.

We included adult patients (≥18 years) admitted to medical or surgical ICUs with ICU length of stay ≥7 days to ensure sufficient observation periods for 7-day trajectory analysis. Patients were excluded if they had missing data on key covariates (age, sex) or the primary outcome (hospital mortality). Fig. 1 presents the patient selection flowchart following STROBE guidelines, including the total number of MIMIC-IV ICU admissions and the number excluded at each step. The final analytical cohort comprised 7,334 patients.

### 2.3 Nursing intensity measurement

Nursing intensity (NI) was operationalized as a daily composite score incorporating three clinically relevant dimensions of observable bedside care demand. Our approach draws conceptually on established nursing workload instruments such as the Nursing Activities Score (NAS; Miranda et al., 2003) and the Therapeutic Intervention Scoring System (TISS-28; Miranda et al., 1996), while using variables directly available in the MIMIC-IV database rather than prospectively collected workload scores. Each dimension was scored on a 0–1 scale:

Dimension 1 – Sedation Management (weight: 1/3): Calculated as 0.5 × (mean absolute RASS score / 5) + 0.5 × (proportion of assessments with RASS ≤ −3). This captures the intensity of sedation monitoring and titration, a core bedside nursing activity (Sessler et al., 2002; Barr et al., 2013). Data source: RASS assessments documented by bedside nurses.
Dimension 2 – Monitoring Frequency (weight: 1/3): Calculated as (hourly charting count / 99th percentile), clipped to [0, 1]. This directly quantifies nursing surveillance activity including vital sign assessments, clinical observations, and documentation. Data source: nursing charting records.
Dimension 3 – Care Complexity (weight: 1/3): Calculated as 0.3 × (mechanical ventilation active) + 0.3 × (vasopressor use) + 0.2 × (renal replacement therapy) + 0.2 × (FiO2 / 100). This captures the complexity of organ support, which generates substantial nursing workload. Data sources: ventilator settings, medication administration records.

The composite NI score was calculated as: NI = (Dim1 + Dim2 + Dim3) / 3 × 100, yielding a continuous score ranging from 0 to 100, with higher scores indicating greater care intensity. The term ‘nursing intensity’ is used here to denote observable bedside care demand that is predominantly delivered and managed by nursing staff, rather than a purely nursing-exclusive construct. The score was calculated for each of ICU Days 1 through 7. We acknowledge that Dimension 3 overlaps with medical treatment intensity; a sensitivity analysis excluding this dimension is reported in Section 3.5.

### 2.4 Illness severity assessment

Illness severity was assessed using the Sequential Organ Failure Assessment (SOFA) score (Vincent et al., 1996) calculated from ICU Day 1 data. The SOFA score ranges from 0 to 24, assessing dysfunction across six organ systems: respiratory, coagulation, hepatic, cardiovascular, neurological, and renal. Higher scores indicate greater severity of organ dysfunction. SOFA scores were derived from the validated MIMIC-IV derived tables (Johnson et al., 2023). The SOFA score was not included as an input to the latent class trajectory modeling to preserve the assumption of local independence; it was used exclusively as a covariate in the subsequent regression models.

### 2.5 Clinical characteristics

Additional clinical characteristics were extracted from the MIMIC-IV database, including age, sex, ICU length of stay, mechanical ventilation status, vasopressor use, and renal replacement therapy (RRT). Mechanical ventilation was defined as invasive ventilation for >24 hours. Vasopressor use included administration of norepinephrine, epinephrine, phenylephrine, vasopressin, or dopamine. RRT included hemodialysis, peritoneal dialysis, or continuous renal replacement therapy.

### 2.6 Statistical analysis

#### 2.6.1 Latent class trajectory modeling

We used Gaussian mixture models (GMM) to identify distinct latent classes of NI trajectories over ICU Days 1–7 (Nagin and Odgers, 2010). Model selection proceeded through systematic comparison of 2- through 6-class solutions. The optimal model was selected based on: (1) Bayesian Information Criterion (BIC; lower values preferred); (2) entropy (higher values indicating better classification quality); (3) average posterior probabilities of class membership (>0.80 for all classes); and (4) clinical interpretability. Complete model comparison results are reported in Supplementary Table S1.

#### 2.6.2 Outcome analysis

The primary outcome was hospital mortality. The secondary outcome was ICU length of stay. For mortality, we constructed three nested multivariable logistic regression models: Model 1 (unadjusted), Model 2 (adjusted for age and sex), and Model 3 (additionally adjusted for SOFA score). Class 4 (‘Persistent High’) served as the reference group as the largest class (61.0%). Odds ratios (OR) and 95% confidence intervals (CI) were calculated. For ICU LOS, we used linear regression of log-transformed LOS with the same adjustment strategy, reporting results as percentage change. Statistical significance was set at p < 0.05.

#### 2.6.3 Supplementary analyses

To assess construct validity and robustness, we conducted: (1) Spearman correlation between mean NI scores and baseline SOFA to evaluate the degree of overlap between nursing intensity and illness severity; (2) sensitivity analysis reconstructing the NI score using only Dimensions 1 and 2 (sedation and monitoring), excluding Dimension 3 (care complexity), to determine whether trajectory classes are robust to exclusion of medical intervention variables; (3) bootstrap validation with 500 resamples to assess class stability, with particular attention to the smallest class; and (4) formal regression analysis of ICU length of stay. All analyses were performed using Python 3.10 (scikit-learn for GMM, statsmodels for regression, scipy for correlation analysis).

## 3 Results

### 3.1 Sample characteristics

Table 1 presents baseline characteristics of the 7,334 patients meeting inclusion criteria. Mean age was 63.0 years (SD 15.6, range 18–89), with 57.9% male. Median ICU length of stay was 12.3 days (IQR: 9.1–18.1). Mean SOFA score on ICU Day 1 was 8.8 (SD 3.7). Nearly all patients (99.5%) required mechanical ventilation, 74.0% received vasopressors, and 19.5% required RRT. Overall hospital mortality was 26.4%.

**Table 1:** Baseline characteristics of study population (N = 7334).

Characteristic	Value
Age, years, mean (SD, range)	63.0 (15.6, 18–89)
Male, n (%)	4,248 (57.9%)
ICU LOS, days, median (IQR)	12.3 (9.1–18.1)
SOFA Day 1, mean (SD)	8.8 (3.7)
Mechanical ventilation, n (%)	7,298 (99.5%)
Vasopressor use, n (%)	5,428 (74.0%)
Renal replacement therapy, n (%)	1,432 (19.5%)
Hospital mortality, n (%)	1,939 (26.4%)

SD, standard deviation; IQR, interquartile range; LOS, length of stay; SOFA, Sequential Organ Failure Assessment. The high prevalence of mechanical ventilation reflects the study’s inclusion criterion of ICU length of stay ≥7 days, selecting patients with severe illness requiring prolonged intensive care.

### 3.2 Nursing intensity trajectory classes

**Fig. 2:**
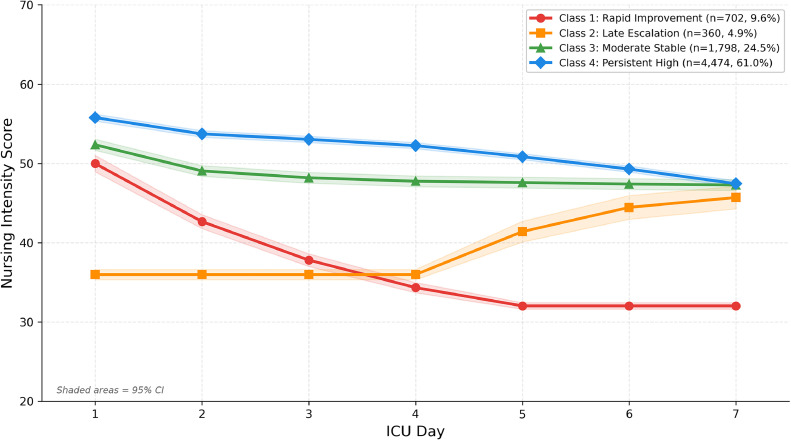
Nursing Intensity trajectories by class over ICU Days 1–7. Four distinct trajectory patterns were identified: Class 1 ‘Rapid Improvement’ (red, n = 702, 9.6%) with steeply declining NI; Class 2 ‘Late Escalation’ (orange, n = 360, 4.9%) with rising NI; Class 3 ‘Moderate Stable’ (green, n = 1,798, 24.5%) with mildly declining NI; and Class 4 ‘Persistent High’ (blue, n = 4,474, 61.0%) with consistently elevated NI. Shaded areas represent 95% confidence intervals. The NI composite score (range 0–100) incorporates sedation management, monitoring frequency, and care complexity.

Latent class trajectory modeling identified four distinct trajectory patterns (Fig. 2). The 4-class model was selected based on lowest BIC (65,766), highest entropy (0.948), and clinically interpretable patterns. Supplementary Table S1 reports fit indices for all candidate models (2–6 classes). Average posterior probabilities exceeded 0.93 for all classes.

Class 1 – ‘Rapid Improvement’ (n = 702, 9.6%): Characterized by steeply declining NI scores from Day 1 (mean 50.0) to Day 7 (mean 32.0), reflecting rapidly decreasing bedside care demand over the first week.
Class 2 – ‘Late Escalation’ (n = 360, 4.9%): Characterized by initially stable NI (Day 1 mean 36.0) that rises from Day 5 onward (Day 7 mean 45.7), indicating increasing care requirements over time.
Class 3 – ‘Moderate Stable’ (n = 1,798, 24.5%): Demonstrated mildly declining NI from Day 1 (mean 52.4) to Day 7 (mean 47.3), with relatively stable care intensity.
Class 4 – ‘Persistent High’ (n = 4,474, 61.0%): Maintained consistently elevated NI throughout the observation period (Day 1 mean 55.8, Day 7 mean 47.5).

We note that two classes fall below the 10% threshold sometimes recommended for latent class retention (Sinha et al., 2020; Sinha et al., 2021). Although classes below 10% are often considered potentially unstable in latent class frameworks, bootstrap validation (500 resamples) confirmed that all classes were reproducible: the smallest class never vanished across all iterations (minimum 3.6% of sample), and 98.2% of resamples achieved ARI > 0.40 with the original solution (Supplementary Table S4). These results suggest that these small classes represent reproducible rare trajectory patterns rather than modeling artifacts.

### 3.3 Characteristics and mortality by trajectory class

**Fig. 3:**
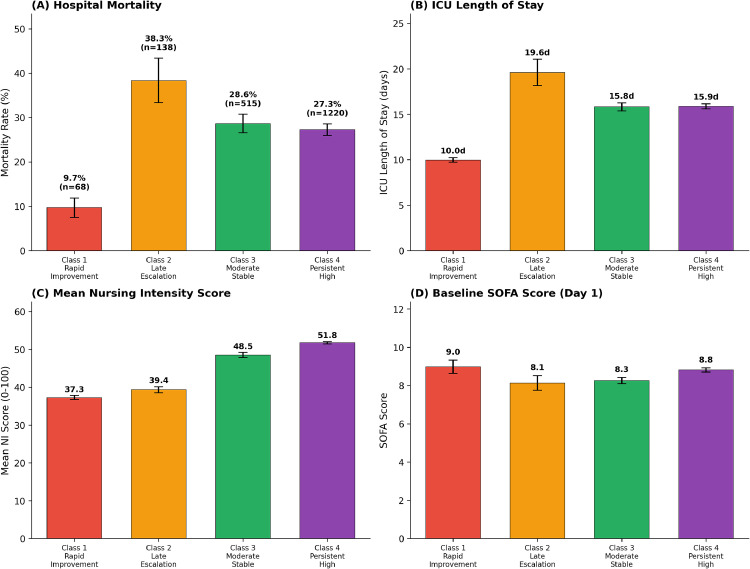
Clinical outcomes and characteristics by trajectory class. (A) Hospital mortality rate with 95% CI; (B) Mean ICU length of stay in days; (C) Mean Nursing Intensity composite score (range 0–100); (D) Baseline SOFA score on ICU Day 1. Despite comparable SOFA scores across classes, mortality and ICU LOS differed substantially, with Class 1 (Rapid Improvement) showing the lowest mortality (9.7%) and shortest ICU stay (10.0 days), while Class 2 (Late Escalation) showed the highest mortality (38.3%) and longest stay (19.6 days). Error bars represent 95% confidence intervals.

Table 2 presents characteristics stratified by trajectory class. SOFA scores were comparable across classes (range 8.2–9.1). Despite similar baseline severity, mortality differed substantially: Class 1 (9.6%), Class 2 (39.3%), Class 3 (28.6%), and Class 4 (27.4%), (Fig. 3).

**Table 2:** Characteristics and outcomes by nursing intensity trajectory phenotype.

Characteristic	Overall	Class 1 Rapid Improvement	Class 2 Late Escalation	Class 3 Moderate Stable	Class 4 Persistent High
n (%)	7,334 (100)	702 (9.6)	360 (4.9)	1,798 (24.5)	4,474 (61.0)
Age, mean ± SD	63.0 ± 15.6	61.5 ± 15.8	63.2 ± 15.0	62.8 ± 15.5	63.4 ± 15.7
Male, %	57.9	58.3	56.9	58.1	57.8
SOFA, mean ± SD	8.8 ± 3.7	8.2 ± 3.5	9.1 ± 3.8	8.5 ± 3.6	8.9 ± 3.7
NI Day 1, mean	—	50.0	36.0	52.4	55.8
NI Day 7, mean	—	32.0	45.7	47.3	47.5
ICU LOS, median (IQR)	12.3 (9.1–18.1)	8.7 (7.7–10.7)	16.6 (12.7–25.4)	13.0 (9.6–18.7)	12.9 (9.4–18.9)
Hospital mortality, %	26.4	9.6	39.3	28.6	27.4

SD, standard deviation; IQR, interquartile range; LOS, length of stay; SOFA, Sequential Organ Failure Assessment; NI, Nursing Intensity. Green shading highlights Class 1 with shortest ICU stay and lowest mortality. Kruskal-Wallis test: SOFA p=0.029; ICU LOS p<0.001.

Table 3 presents multivariable logistic regression results. In the fully adjusted model (Model 3), Class 1 (Rapid Improvement) was associated with substantially lower mortality compared to Class 4 (OR = 0.26, 95% CI: 0.18–0.36, p < 0.001), and Class 2 (Late Escalation) with higher mortality (OR = 1.86, 95% CI: 1.45–2.39, p < 0.001). Class 3 did not differ significantly from Class 4 (OR = 1.09, 95% CI: 0.96–1.24, p = 0.17).

**Table 3:** Multivariable logistic regression predicting hospital mortality (Reference: Class 4).

Class	Model 1 OR (95% CI)	p	Model 2 OR (95% CI)	p	Model 3 OR (95% CI)	p
1 Rapid Improvement	0.28 (0.20–0.39)	<0.001	0.26 (0.19–0.37)	<0.001	0.26 (0.18–0.36)	<0.001
2 Late Escalation	1.72 (1.35–2.19)	<0.001	1.76 (1.37–2.25)	<0.001	1.86 (1.45–2.39)	<0.001
3 Moderate Stable	1.06 (0.94–1.20)	0.326	1.05 (0.93–1.19)	0.454	1.09 (0.96–1.24)	0.166

Model 1: unadjusted. Model 2: adjusted for age and sex. Model 3: adjusted for age, sex, and SOFA score. OR, odds ratio; CI, confidence interval.

Table 4 presents the complete Model 3 regression results, including the effects of all covariates. Each year of age was associated with 2% higher mortality odds (OR = 1.02, 95% CI: 1.02–1.03, p < 0.001). Male sex showed a trend toward lower mortality (OR = 0.91, 95% CI: 0.81–1.01, p = 0.084). Each additional SOFA point was associated with 7% higher odds (OR = 1.07, 95% CI: 1.06–1.09, p < 0.001). Importantly, trajectory class associations remained significant after adjustment for these covariates, confirming independent prognostic value.

**Table 4:** Complete multivariable logistic regression results for all trajectory phenotypes (Model 3).

Comparison	OR (95% CI)	p-value
Class 1 vs Class 4	0.26 (0.18–0.36)	<0.001
Class 2 vs Class 4	1.86 (1.45–2.39)	<0.001
Class 3 vs Class 4	1.09 (0.96–1.24)	0.166
Class 4 vs Class 4	1.00 (Reference)	—
Covariates		
Age (per year)	1.02 (1.02–1.03)	<0.001
Male gender	0.91 (0.81–1.01)	0.084
SOFA score (per point)	1.07 (1.06–1.09)	<0.001

Model 3 adjusted for age, sex, and SOFA score. OR, odds ratio; CI, confidence interval; SOFA, Sequential Organ Failure Assessment. Class 4 (Persistent High) serves as the reference group (largest class, 61.0%). Green shading highlights the key finding (Class 1 protective association).

### 3.4 ICU length of stay analysis

Linear regression of log-transformed ICU LOS, adjusted for age, sex, and SOFA score, revealed significant differences across trajectory classes (Supplementary Table S5). Compared to Class 4, Class 1 (Rapid Improvement) was associated with 32.0% shorter ICU stays (95% CI: −35.3% to −28.7%, p < 0.001), while Class 2 (Late Escalation) was associated with 30.0% longer stays (95% CI: +22.9% to +37.4%, p < 0.001). Class 3 did not differ significantly from Class 4 (+0.2%, p = 0.87).

### 3.5 Supplementary analyses

#### 3.5.1 NI–SOFA correlation

Mean NI scores showed weak correlation with baseline SOFA scores (Spearman ρ = 0.142, p < 0.001; n = 6,905). Examining individual dimensions, Dimension 1 (sedation) showed a negative correlation (ρ = −0.101), Dimension 2 (monitoring) a weak positive correlation (ρ = 0.152), and Dimension 3 (care complexity) a moderate positive correlation (ρ = 0.375) with SOFA (Supplementary Table S2). These findings support the interpretation that the NI score captures dimensions of care demand that are largely independent of baseline illness severity as measured by SOFA.

#### 3.5.2 Sensitivity analysis excluding medical interventions

When the NI score was reconstructed using only Dimensions 1 (sedation) and 2 (monitoring frequency), excluding Dimension 3 (care complexity), the 4-class GMM yielded trajectory patterns with moderate–strong agreement with the original classification (Adjusted Rand Index = 0.565). The sensitivity classes preserved the mortality gradient and showed similar trajectory shapes (Supplementary Table S3). This confirms that the identified trajectory patterns are primarily driven by nursing-specific assessments rather than by medical intervention variables.

#### 3.5.3 Bootstrap validation

Bootstrap validation with 500 resamples demonstrated good reproducibility of the 4-class solution. The mean Adjusted Rand Index between original and bootstrap classifications was 0.613 (95% CI: 0.411–0.973), with 98.2% of iterations achieving ARI > 0.40. The smallest class never vanished across all 500 resamples (minimum 3.6% of sample; Supplementary Table S4).

## 4 Discussion

### 4.1 Principal findings

This study identified four distinct nursing intensity trajectory patterns in 7,334 ICU patients with prolonged stays (≥7 days). The key finding is that trajectory class membership was independently associated with hospital mortality after adjustment for age, sex, and baseline illness severity (SOFA score). Patients exhibiting a ‘Rapid Improvement’ trajectory—characterized by steeply declining NI scores over the first 7 ICU days—had 74% lower odds of hospital mortality compared to those with ‘Persistent High’ NI (OR = 0.26, 95% CI: 0.18–0.36). Conversely, patients with a ‘Late Escalation’ pattern—rising NI over the first week—had 86% higher mortality odds (OR = 1.86, 95% CI: 1.45–2.39).

Importantly, these findings should be interpreted as reflecting care-response patterns rather than causal effects of nursing intensity on outcomes. The observed associations likely capture the interplay between patient physiology and care delivery: declining NI may reflect clinical improvement enabling care de-escalation, while escalating NI may indicate clinical deterioration requiring intensified support.

### 4.2 Interpretation of trajectory patterns

The ‘Rapid Improvement’ trajectory (Class 1, 9.6%) is notable for its combination of declining care intensity, shortest ICU stay (median 8.7 days), and lowest mortality (9.6%), despite comparable baseline SOFA scores. This pattern is consistent with a subgroup exhibiting favorable clinical trajectories during the early ICU course, in whom progressive weaning of monitoring, sedation, and organ support was feasible. It is important to note that even the ‘low’ end of this trajectory still represents substantial ICU nursing care; the clinically meaningful distinction is the direction and rate of change rather than absolute intensity levels.

The ‘Late Escalation’ trajectory (Class 2, 4.9%) is the smallest but clinically most concerning class, characterized by initially moderate NI that rises over the week. These patients had the highest mortality (39.3%) and longest ICU stays (median 16.6 days), suggesting a subgroup that deteriorates despite ongoing intensive care. This pattern may represent patients developing secondary complications such as nosocomial infections, organ failure progression, or treatment-resistant conditions.

The weak correlation between NI and SOFA scores (ρ = 0.14) provides empirical evidence that these trajectory patterns capture dimensions of care demand beyond illness severity alone. The sensitivity analysis excluding medical intervention variables (ARI = 0.565) further confirms that the patterns are primarily driven by nursing-specific assessments—sedation management and monitoring frequency—rather than by medical treatment decisions. Nevertheless, we acknowledge that the identified classes may partly reflect evolving illness burden, and that the NI score captures observable bedside care demand rather than a purely nursing-specific construct.

### 4.3 Reverse causality and temporal considerations

A critical consideration is reverse causality: declining NI trajectories may reflect clinical improvement driving care de-escalation rather than low care intensity promoting recovery. Trajectory class membership should therefore be interpreted as a descriptor of evolving illness–care coupling rather than an independent exposure with a direct causal effect on outcomes. In the absence of time-lagged models or marginal structural models, we cannot disentangle whether trajectory patterns drive outcomes or merely reflect underlying disease trajectories. The temporal structure of our study—with severity measured at baseline (Day 1) and trajectories classified over Days 1–7—provides a framework for characterizing these patterns, but temporal ordering alone does not establish causal direction. Time-varying confounding and intertwined disease progression remain important limitations. Future prospective studies with time-lagged designs are needed to address these questions.

### 4.4 Clinical implications

These findings have several potential implications, though all require prospective validation before informing practice. First, the existence of distinct trajectory classes with different outcome profiles suggests that patients may have heterogeneous responses to ICU care that become apparent over the first week. Second, the substantial difference in ICU length of stay between classes (median 8.7 vs. 16.6 days) suggests potential relevance for resource planning. Third, if trajectory patterns can be predicted early—for example, within the first 48–72 hours—this could eventually inform individualized care intensity adjustments.

However, we emphasize that latent class trajectory modeling is inherently retrospective. Prospective identification of class membership would require the development and validation of early prediction models using readily available clinical indicators, which represents an important direction for future research. The current study provides the descriptive foundation for such translational work rather than immediately actionable staffing recommendations.

### 4.5 Limitations

This study has several limitations. First, the ≥7-day ICU LOS requirement introduces survivorship bias by excluding patients who died early or improved rapidly and were discharged before Day 7. This restricts our findings to a prolonged-stay, high-acuity subpopulation that may not represent ICU patients broadly. The average US ICU length of stay is 3–4 days (Kannan et al., 2024), making our cohort a distinct subgroup. Second, this is a single-center study from a tertiary academic medical center and Level I trauma center that treats sicker and more complex patients than the average US hospital; findings may not generalize to community hospitals or different healthcare systems. Third, the NI composite score, while incorporating nursing-relevant dimensions, also includes variables that overlap with medical treatment intensity (Dimension 3); our sensitivity analysis excluding these variables partially addresses this concern. Fourth, residual confounding from unmeasured variables (e.g., comorbidity burden, surgical vs. medical diagnosis, pre-ICU functional status) cannot be excluded in this observational design. Fifth, we could not assess nurse staffing levels, skill mix, or nursing expertise, which may independently influence both NI patterns and outcomes. Sixth, the near-universal mechanical ventilation rate (99.5%) reflects the high-acuity nature of this selected cohort and limits generalizability to less severely ill ICU populations. Seventh, although our observational design cannot establish causality, it provides a foundation for hypothesis generation regarding trajectory-guided approaches to ICU nursing care.

## 5 Conclusions

Using latent class trajectory modeling in a large cohort of 7,334 ICU patients with prolonged stays, we identified four distinct nursing intensity trajectory patterns over the first 7 ICU days. These patterns showed independent associations with hospital mortality after adjustment for baseline illness severity: ‘Rapid Improvement’ trajectories characterized patients with lower mortality and shorter ICU stays, while ‘Late Escalation’ trajectories were observed in patients with higher mortality and longer stays. The NI score demonstrated weak correlation with illness severity, and trajectory patterns remained robust when medical intervention variables were excluded. These observational findings provide a descriptive foundation for future prospective research exploring whether early identification of nursing intensity trajectory patterns could inform individualized care approaches in critical care settings.

## Funding

This research received no specific grant from any funding agency in the public, commercial, or not-for-profit sectors.

## Data source

The data that support the findings of this study are available from the Medical Information Mart for Intensive Care (MIMIC-IV) database. MIMIC-IV is a publicly available database containing de-identified health data associated with patients admitted to intensive care units at Beth Israel Deaconess Medical Center between 2008 and 2019.

## Data access

The MIMIC-IV database is freely available to researchers worldwide. Access requires:

1. Completion of the Collaborative Institutional Training Initiative (CITI) "Data or Specimens Only Research" course.
2. Signing a data use agreement.
3. Registration on PhysioNet (https://physionet.org/).

Detailed instructions for obtaining access are available at: https://mimic.mit.edu/docs/gettingstarted/.

## Data use

This study used MIMIC-IV version 2.2. The specific dataset extraction and analysis code used in this study can be made available upon reasonable request to the corresponding author, subject to the data use agreement restrictions of the MIMIC-IV database.

## Ethical approval

The use of MIMIC-IV data for this research was deemed exempt from institutional review board approval as it involves analysis of publicly available, de-identified data. The original data collection for MIMIC-IV was approved by the Institutional Review Boards of Beth Israel Deaconess Medical Center and the Massachusetts Institute of Technology.

## For questions regarding data

For questions regarding the data used in this study, please contact: Yan Li, PhD, Email: criticalcare@163.com, Department of Emergency Medicine, Shanghai Fourth People's Hospital, Tongji University.

## CRediT authorship contribution statement

**Qinqin Shu:** Methodology, Data curation. **Yan Li:** Writing – review & editing, Resources, Project administration.

## Declaration of competing interest

The authors declare that they have no known competing financial interests or personal relationships that could have appeared to influence the work reported in this paper.

## Appendix Supplementary materials

Image, application 1
