# Supplementary material for: Nursing intensity trajectory patterns and clinical outcomes in intensive care units: a latent class analysis
Source: Int J Nurs Stud Adv. 2026 Jun 26;11:100613. doi: 10.1016/j.ijnsa.2026.100613 (PMC13332457; doi:10.1016/j.ijnsa.2026.100613)
Supplement: Supplementary file 1 [file mmc1.docx]

**Supplementary Materials**

IJNSA-D-26-00071 (Revised)

## **Supplementary Table S1. Latent Class Model Comparison (2–6 Classes)**

| **Classes** | **BIC** | **AIC** | **Entropy** | **Class Sizes** | **Avg Posterior Prob** |
| --- | --- | --- | --- | --- | --- |
| 2 | 86,477 | 85,988 | 0.887 | 4,890 (66.7%) / 2,444 (33.3%) | 0.981 / 0.943 |
| 3 | 74,801 | 74,063 | 0.934 | 4,803 (65.5%) / 1,797 (24.5%) / 734 (10.0%) | 0.981 / 0.933 / 0.998 |
| 4* | 65,766 | 64,780 | 0.948 | 4,474 (61.0%) / 1,798 (24.5%) / 702 (9.6%) / 360 (4.9%) | 0.981 / 0.930 / 0.994 / 0.999 |
| 5 | 68,095 | 66,860 | 0.886 | 3,563 / 1,397 / 1,385 / 579 / 410 | 0.962 / 0.894 / 0.880 / 0.883 / 1.000 |
| 6 | 60,245 | 58,761 | 0.886 | 3,415 / 1,325 / 970 / 899 / 409 / 316 | 0.957 / 0.901 / 0.840 / 0.826 / 1.000 / 1.000 |

** Selected model. BIC, Bayesian Information Criterion; AIC, Akaike Information Criterion. The 4-class model achieved the best balance of model fit (lowest BIC), classification quality (highest entropy), and clinical interpretability. Although the 6-class model had lower AIC, its entropy dropped and several classes showed overlapping trajectory patterns with limited clinical distinction.*

## **Supplementary Table S2. Correlation Between Nursing Intensity and SOFA Score**

| **Comparison** | **Coefficient** | **p-value** | **n** |
| --- | --- | --- | --- |
| Mean NI vs SOFA (Pearson r) | 0.143 | 8.03 × 10⁻³³ | 6,905 |
| Mean NI vs SOFA (Spearman ρ) | 0.142 | 1.23 × 10⁻³² | 6,905 |
| Day-1 NI vs SOFA (Spearman ρ) | 0.160 | 7.12 × 10⁻⁴¹ | 6,905 |
| Dim 1 (Sedation) vs SOFA (ρ) | −0.101 | 3.98 × 10⁻¹⁷ | 6,905 |
| Dim 2 (Monitoring) vs SOFA (ρ) | 0.152 | 6.15 × 10⁻³⁷ | 6,905 |
| Dim 3 (Complexity) vs SOFA (ρ) | 0.375 | 5.26 × 10⁻²³⁰ | 6,905 |

*NI, Nursing Intensity; SOFA, Sequential Organ Failure Assessment. The weak overall correlation (r = 0.14) indicates that NI captures dimensions of care demand largely independent of illness severity. Dim 3 (care complexity) shows the strongest correlation (ρ = 0.38) as expected, since both NI Dim 3 and SOFA reflect organ support requirements. Dim 1 (sedation) is negatively correlated, suggesting deeper sedation is not simply a marker of greater illness severity.*

## **Supplementary Table S3. Sensitivity Analysis: NI Score Excluding Medical Interventions**

The NI score was reconstructed using only Dimension 1 (sedation management) and Dimension 2 (monitoring frequency), excluding Dimension 3 (care complexity). A 4-class GMM was fitted to this reduced score.

| **Metric** | **Value** |
| --- | --- |
| Adjusted Rand Index (original vs sensitivity) | 0.565 |
| Patients compared | 7,334 |
| Original NI: mean (SD) | 49.0 (14.7) |
| Sensitivity NI: mean (SD) | 43.9 (16.6) |

Sensitivity Class Trajectories and Mortality:

| **Sensitivity Class** | **n** | **Mortality** | **Day 1 Mean** | **Day 7 Mean** | **Pattern** |
| --- | --- | --- | --- | --- | --- |
| 1 (Persistent High) | 567 | 51.0% | 66.2 | 65.9 | High stable |
| 2 (Rapid Decline) | 549 | 15.8% | 47.2 | 39.3 | Declining |
| 3 (Gradual Decline) | 4,548 | 26.8% | 52.4 | 41.2 | Gradual decline |
| 4 (Moderate Decline) | 1,670 | 20.7% | 40.5 | 31.6 | Moderate decline |

*ARI = 0.565 indicates moderate–strong agreement, confirming that trajectory patterns are primarily driven by nursing-specific dimensions (sedation and monitoring) rather than medical intervention variables. The mortality gradient is preserved in the sensitivity analysis.*

## **Supplementary Table S4. Bootstrap Validation of Class Stability**

| **Metric** | **Value** |
| --- | --- |
| Number of bootstrap iterations | 500 |
| Mean ARI (original vs bootstrap) | 0.613 |
| Median ARI | 0.523 |
| 95% CI of ARI | [0.411, 0.973] |
| Iterations with ARI > 0.60 | 26.4% |
| Iterations with ARI > 0.40 | 98.2% |
| Smallest class: mean size | 8.5% |
| Smallest class: 95% CI | [3.8%, 11.2%] |
| Smallest class: minimum size | 3.6% |
| Smallest class vanishes (<1%) | Never (0/500 iterations) |

Class Size Stability (sorted by rank):

| **Rank** | **Mean %** | **95% CI** |
| --- | --- | --- |
| 1 (Largest) | 52.6% | [46.0%, 62.1%] |
| 2 | 22.2% | [20.0%, 25.2%] |
| 3 | 16.7% | [9.5%, 21.4%] |
| 4 (Smallest) | 8.5% | [3.8%, 11.2%] |

*ARI, Adjusted Rand Index. Bootstrap resampling with replacement (n = 500) was performed to assess the reproducibility of the 4-class solution. Mean ARI of 0.613 indicates good agreement. The smallest class never vanished in any bootstrap sample, confirming it represents a stable subgroup rather than a statistical artifact.*

## **Supplementary Table S5. ICU Length of Stay Regression (Reference: Class 4)**

Linear regression of log-transformed ICU LOS. Results are reported as percentage change in LOS relative to Class 4 (Persistent High).

| **Comparison** | **Unadjusted % Change** | **p** | **Adjusted % Change (95% CI)** | **p** |
| --- | --- | --- | --- | --- |
| Class 1 vs 4 | −32.3% | <0.001 | −32.0% (−35.3% to −28.7%) | <0.001 |
| Class 2 vs 4 | +30.3% | <0.001 | +30.0% (+22.9% to +37.4%) | <0.001 |
| Class 3 vs 4 | +0.3% | 0.831 | +0.2% (−2.4% to +2.9%) | 0.871 |

Covariate effects in adjusted model:

| **Covariate** | **% Change (95% CI)** | **p** |
| --- | --- | --- |
| Age (per year) | −0.1% (−0.2% to −0.0%) | 0.002 |
| Male sex | +1.4% (−0.8% to +3.8%) | 0.215 |
| SOFA (per point) | −0.3% (−0.6% to −0.0%) | 0.046 |

*Adjusted model includes age, sex, and SOFA score. R² = 0.052 (adjusted model). Class 1 patients had 32% shorter ICU stays and Class 2 patients 30% longer stays compared to Class 4, independent of age, sex, and illness severity. The modest R² indicates that trajectory class membership explains a small but statistically significant proportion of LOS variance, consistent with the multifactorial nature of ICU discharge decisions.*
